# Surgical Results of Phacoemulsification Performed by Residents: A Time-Trend Analysis in a Teaching Hospital from 2005 to 2021

**DOI:** 10.1155/2022/4721904

**Published:** 2022-04-25

**Authors:** Jiahn-Shing Lee, Chiun-Ho Hou, Ken-Kuo Lin

**Affiliations:** Department of Ophthalmology, Chang Gung Memorial Hospital and College of Medicine, Chang Gung University, Taoyuan, Taiwan

## Abstract

**Purpose:**

To report a long-term trend of surgical results of phacoemulsification performed by residents in a teaching hospital.

**Methods:**

This study analyzed 1,409 consecutive cases of phacoemulsification performed by residents under a single supervisor from July 2005 to March 2021. The 15.75-year period was divided into seven equal intervals for time-trend analysis. *Main Outcome Measures*. Rates of completion and complications were collected to assess the surgical results.

**Results:**

The overall completion rate was 60.5% (852/1409), and the intraoperative complication rate was 14.5% (204/1409). The completion rates from the first to the seventh interval were 44.7%, 54.2%, 60.6%, 50.6%, 65.1%, 72.5%, and 81.8%, respectively. The completion rate improved significantly with time, mainly in the steps of anterior capsulorhexis and nucleus emulsification. The intraoperative complication rates from the first to the seventh interval were 27.4%, 20.4%, 14.0%, 11.8%, 8.2%, 9.6%, and 7.3%, respectively. The complication rate also decreased significantly with time, mainly in the steps of anterior capsulorhexis, nucleus emulsification, and cortex removal. The major complications included anterior capsulorhexis tear (*n* = 95), vitreous loss (*n* = 40), iris damage or prolapse (*n* = 36), and posterior capsule tear without vitreous loss (*n* = 29). There was a significant improvement of surgical results with the level of residency in the completion rate but not in the complication rate.

**Conclusions:**

With a long-term evolution in the surgical training curriculum, it is possible to reach a goal of both higher completion and lower complication rates of resident-performed phacoemulsification.

## 1. Introduction

Cataract surgery with phacoemulsification is a challenging skill both to learn and to teach [1, 2]. To prevent or to manage intraoperative complications, a supervisor surgeon may have to take over part of the surgery during the surgical process. However, takeover rates were rarely reported in previous studies. Wide variability, ranging from 12% to 75%, had been reported in the few studies on completion and complication rates [3–6]. It is infeasible for a retrospective study to obtain residents' completion rate of phacoemulsification; hence, prospective studies with well-defined criteria for takeover are needed before meaningful comparisons can be made [1].

Our previous study prospectively documented our residents' surgical results with definite criteria for the trainer's takeover [6]. The findings showed an exponential pattern in the “learning curve” of resident-performed phacoemulsification. In view of the need not only to decrease the residents' complication rate but also to increase their completion rate, this research extended our previous study period from 2.25 years (226 cases) to 15.75 years (1409 cases). A longer observation period would on the one hand reveal an institutional evolution in training results of phacoemulsification, and on the other hand gain insight into the “teaching curve” of our trainers.

## 2. Methods

This study collected surgical results, prospectively registered, of 1,409 consecutive cases of phacoemulsification (phaco) performed by 63 resident ophthalmologists under a single supervisor (JS Lee) from July 2005 to March 2021 at Chang Gung Memorial Hospital (CGMH) in Taiwan. This 15.75-year period was divided into seven equal intervals for a time-trend analysis, with 2.25 years in each interval; that is, the first interval was from July 2005 to September 2007, while the seventh or final interval was from January 2019 to March 2021. The training details were similar to those in our previous study with a small variation in the distribution of the level of residency [6]. In this study, 515 cases (36.6%) were performed by fourth-year residents (R4), 766 cases (54.4%) by third-year residents (R3), 113 cases (8.0%) by second-year residents (R2), and 15 cases (1.1%) by first-year residents (R1). Although our junior residents (R1 and R2) generally received “part-phaco or step-by-step” training, they had “full-phaco or one-step” training if they could in this study [6, 7].

The criteria for the supervisor to take over included three failed attempts of any maneuver, anterior capsulorhexis extending to the iris, repeated iris prolapse, or at resident's or attending's discretion [6]. As long as no major complication occurred during the surgical process, the resident would complete the entire operation. Intraoperative complications, if occurred, and any of the following four outcomes, namely, (A) incomplete surgery due to supervisor's interference and without complications, (B) incomplete surgery with either major or minor complications, (C) complete surgery with minor complications, and (D) complete surgery without complications, were recorded immediately after the operation. The “completion rate” is calculated as the number of cases with (C) + (D) outcomes divided by the total number of cases (A) + (B) + (C) + (D), and the “complication rate” equals the number of cases with (B) + (C) outcomes divided by the total number of cases. Complications that occurred when the supervisor performed the surgical process were excluded from the residents' complication rate. Group differences were determined using Chi-squared tests. A *p* value of 0.05 denoted statistical significance.

This study has obtained approval of exemption from the Institutional Review Board of Chang Gung Memorial Hospital, Taiwan (201700226B0).

## 3. Results

Of the 1,409 phacoemulsification surgeries, our residents independently completed 852 cases (60.5%), in which 37 cases had minor complications, including mild iris damage or prolapse and anterior capsulorhexis tear found after emulsification of nucleus. Of the remaining 557 incomplete cases, our residents encountered a major complication leading to discontinuation of the surgery in 155 cases. The supervisor took over in the other 402 cases, of which 12 cases had minor complications before the takeover. Of the 204 cases with complications (14.5%), 40 cases were complicated with vitreous loss, representing an overall rate of 2.8% (40/1, 409).

**Figure 1:**
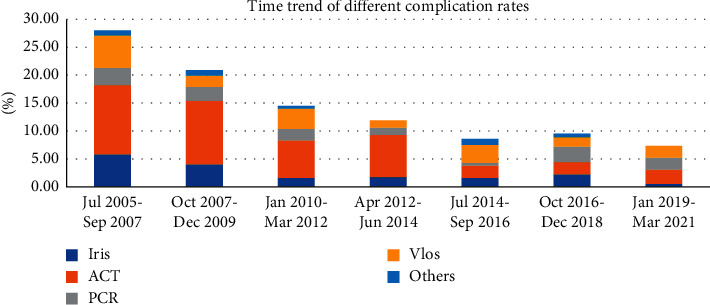
Time trend of different complications of phacoemulsification performed by resident surgeons. Iris: iris damage or prolapse (*p*=0.012), ACT: anterior capsulorhexis tear (*p* < 0.001), PCR: posterior capsule tear without vitreous loss (*p*=0.585), Vlos: vitreous loss (*p*=0.076), PCR + Vlos (*p*=0.075) by Chi-squared test.

In the 204 complicated cases, 208 complication events occurred. Categories of complication events included anterior capsulorhexis tear (95 cases), vitreous loss (40 cases with 3 cases of lens drop), iris damage or prolapse (36 cases), posterior capsule tear without a vitreous loss (29 cases), and others (3 cases of zonular dehiscence, 2 cases of corneal injury, 2 cases of intraocular lens injury or upside down, and 1 choroidal detachment). The time trend of different complications is displayed in Figure 1. The improved complication rate with time was significant only for anterior capsulorhexis tear (*p* < 0.001) and iris damage or prolapse (*p*=0.012).

The time trend for the surgical result of phacoemulsification performed by our residents is shown in Table 1. The completion rate gradually improved from 44.7% in the first interval to 81.8% in the final interval (*p* < 0.001), except for a brief drop from 60.6% in the third interval to 50.6% in the fourth interval. The complication rate also decreased from 27.4% in the first interval to 7.3% in the final interval (*p* < 0.001).

The time trend of completion rates in different steps of phacoemulsification is shown in Table 2. As in our previous study, phacoemulsification involves five steps, including (A) wound incision, (B) anterior capsulorhexis, (C) hydrodissection, hydrodelineation, and emulsification of nucleus, (D) irrigation and aspiration of cortex, and (E) intraocular lens (IOL) insertion. The improved completion rate with time was significant only for steps B and C. The time trend of complication rates in different steps of phacoemulsification is shown in Table 3. The improved complication rate with time was significant for steps B, C, and D.

**Figure 2:**
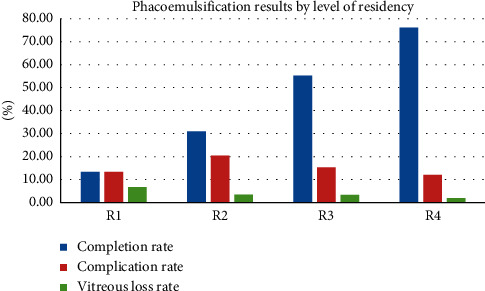
Surgical results of phacoemulsification by the level of residency.

Nearly 90% of the surgeries were performed by our senior residents (R3 and R4), and only 128 cases were done by the juniors (R1 and R2). It was noteworthy that the junior residents performed most of the surgeries (45 cases) in the fourth interval (19.0% of the 237 cases), followed by 32 cases in the fifth interval (17.6% of the 182 cases). The overall completion rates of surgeries performed by R1 to R4 were 13.3% (2/15), 31.0% (35/113), 55.2% (423/766), and 76.1% (392/515), respectively (*p* < 0.001). Intraoperative complication rates in surgeries performed by R1 to R4 were 13.3% (2/15), 20.4% (23/113), 15.3% (117/766), and 12.0% (62/515), respectively (*p*=0.110). Vitreous loss rates in surgeries performed by R1 to R4 were 6.7% (1/15), 3.5% (4/113), 3.3% (25/766), and 1.9% (10/515), respectively (*p*=0.391) (Figure 2).

## 4. Discussion

Cataract surgery is the most performed operation in ophthalmology, and phacoemulsification is a key technique learned by residents in the recent three decades. However, it is challenging for both trainers and trainees due to its distinct property of small error margin and patients' high expectation of both short operation time and immediate visual recovery. Moreover, it is usually performed under topical anesthesia with patients being conscious, thus posing a problem to verbal communication between a trainer and trainee during surgery. A review of teaching methods demonstrated that complication rates decline after a resident performs an average of 70 surgeries [1]. However, there seemed to be a disparity regarding the amount of surgical training between trainers and trainees. For example, a survey of the US program directors revealed that 97% of their residents could complete at least 50 cataract surgeries by the end of the residency training [8]. Another survey of the UK residents disclosed that only 42% of the senior house officers could meet the minimum requirement of 50 complete intraocular operations [9]. In this study, the supervisors would regard all or most of the 1409 surgeries as “full-phaco” training cases. In contrast, the residents might only count the 852 “complete” cases when calculating their surgical experience. In view of such differences, both completion and complication rates should be counted as equally important when evaluating residents' surgical results.

Surgical results of resident-performed phacoemulsification show wide variation due to numerous variables, such as the amount of practice, inherent manual dexterity, and prior experience [1]. Although some creative and innovative methods, such as practicing in the lab or using simulators, adopting a modular training model, and properly assessing surgical competency in the training programs, have been suggested to help trainee surgeons overcome their barrier steps, it was estimated by the program directors that about 10% of the US residents were still recognized as having trouble mastering surgical skills by the end of their residency [10]. With more and more medical students interested in enrolling in the ophthalmology resident training program, it is likely that the academic competence of our recent residents is slightly higher than that of the previous ones. However, academically competent does not necessarily mean surgically skilled, and we estimate that the “quality” of our residents has only a minor effect on improvement in their surgical results of phacoemulsification in this study.

The surgical results were also reported to be affected by the experience of attendings, with the residents' vitreous loss rate much lower under the supervision of an 11-year-seniority attending than a novice [11]. From the beginning of this study, the supervisor already had 13 years of surgical experience in phacoemulsification, but not the novice attending. We believe that the attitude and methods of the supervisor toward the residents' surgical training of phacoemulsification remained consistent from the beginning to the end of this study. The supervisor's experience is mainly used for determining when to take over an unsmooth operation, like a coach asking to pause an ongoing game. The trainees' surgical result is either a low completion rate if taking over too early or a high complication rate if too late. An experienced trainer may decrease the trainees' complication rate but can hardly increase their completion rate simultaneously, and vice versa. Therefore, the experience of the attending had the least effect on the improvement of surgical results of our residents in this study, taking account of both completion and complication rates. Furthermore, we only used a single supervisor's data to exclude possible intertrainer bias.

A structured surgical curriculum program has also been demonstrated to improve the residents' surgical outcomes [12]. Our institute has upgraded the phaco-machine, operative microscopy with video-recording, and the simulating training kits and shortened the wound size in this 15.75-year period. Moreover, the junior residents performed 30–70 capsulorhexis while working with the institute's highest-volume surgeon, and some high-volume surgeons after becoming section or department chief could afford more cases for the residents. Year by year, our attendings, including the authors, also gained experience in both surgical skills and surgical education. These could not occur in just one or two years, but all contributed to the progress in resident-performed phacoemulsification. It is likely that this factor, compared with the above factors of trainers and trainees, makes the most contribution to the improvement of our residents' surgical results. Previous reports investigating the competency of surgical curriculum programs were relatively short in duration. Both the improvement of the surgical curriculum program and its persistence are equally important. To the best of our knowledge, this study had the longest duration of follow-up in phacoemulsification training.

On average, we have three to four new residents each year. All attendings who perform cataract surgery would choose suitable cases and timings to teach phacoemulsification skills for residents according to their own criteria. Nevertheless, DOPS (direct observation of procedural skills) was commonly used for evaluation and to provide feedback for our residents. The feedback is always done immediately after the operation, and operative microscopy with video recording is very helpful for such feedback. The program director changed three times in this study period, monitoring the quality and quantity of surgical training by assessing the DOPS results. The wet lab was held at least once a year, and we had 10 sets of simulating training kits for residents to practice and improve their barrier steps of phacoemulsification.

Although the overall complication rate in this study (14.5%) was lower than that of our previous study (27.4%), the top three complications were the same in both studies. However, contrary to the ranking of complications in this study, the ranking in the previous study was anterior capsulorhexis tear (28 cases) followed by iris complication (13 cases) and vitreous loss (11 cases) [6]. The lower ranking of iris problems in this study is likely related to the shortened incision wound introduced in the middle of this study. Although anterior capsulorhexis tear remained the most frequent complication, it decreased significantly with time in this study, possibly due to our residents' having chances of performing a high volume of capsulorhexis during junior years. Instead of anterior capsulorhexis tear and iris issues being our two major complications, the most frequently reported complications in resident-performed phacoemulsification are posterior capsule rent and vitreous loss [1, 13–15]. Anterior capsulorhexis tear and iris issues, if properly managed, have a less severe effect on the outcome than posterior capsule rent and vitreous loss and are probably under-reported. Yet they are common problems in our resident-performed phacoemulsification and may increase difficulty in completing the remaining surgery if occurred.

The two most difficult phacoemulsification steps reported by basic trainees were anterior capsulorhexis and nucleus emulsification, which also had the lowest completion rates (74.4 and 66.7%, respectively) [16]. Despite the highest rate of supervisor's assistance as reflected by the lowest completion rate in these two steps, our residents' highest complication rate also occurred during anterior capsulorhexis and nucleus emulsification. In a patient-based practice, most supervisors will try their best to avoid a high complication rate of the trainee surgeon and may take over the barrier steps especially for residents who are struggling before the inflection point of their learning process. Arranging junior residents to perform more surgeries of capsulorhexis and to practice in a wet lab or with simulating training kits is helpful for overcoming this problem.

For our time-trend analysis (Table 1), both completion and complication rates improved significantly with time. However, for resident-level analysis (Figure 2), only completion rate, but neither complication nor vitreous loss rates, improved significantly with the residents' level. A possible explanation is that the cases performed by junior residents and those of vitreous loss are too few to reach statistical significance. Another reason may be that we did not control the level of difficulty of the surgery, which is hard to control. It was estimated that only 22.9% of cataract cases were suitable for operation by junior ophthalmologists [17]. Once the residents' surgical skills reached a certain level, most attendings would choose cases of higher difficulty for them. In such circumstances, the trainees' complication rate or vitreous loss rate may not improve as much as the completion rate. A recent European study also had a similar result that the complication rate of resident trainees did not differ according to their level of residency [18].

The key strengths of this study include the large numbers of patients and residents involved, very long study period, prospective records with well-defined criteria, and elimination of the intertrainer bias when assessing institutional surgical training results of phacoemulsification. The outcome is encouraging, but such progress is multifactorial. Our limitation lies in the difficulty to tell exactly which factors are more important. Further studies are necessary to clarify these issues.

## 5. Conclusion

Our results demonstrate that it is possible to reach a goal of both higher completion and lower complication rates of resident-performed phacoemulsification after a long-term evolution in the surgical training curriculum.

## Figures and Tables

**Table 1:** Time trend of completion and complication rates of phacoemulsification performed by residents.

	Jul 2005–Sep 2007	Oct 2007–Dec 2009	Jan 2010–Mar 2012	Apr 2012–Jun 2014	Jul 2014–Sep 2016	Oct 2016–Dec 2018	Jan 2019–Mar 2021	*p* value
Total number of cases	226	201	193	237	182	178	192	
Completion rate (cases)	44.7% (101)	54.2% (109)	60.6% (117)	50.6% (120)	65.1% (119)	72.5% (129)	81.8% (157)	<0.001
Complication rate (cases)	27.4% (62)	20.4% (41)	14.0% (27)	11.8% (28)	8.2% (15)	9.6% (17)	7.3% (14)	<0.001

*p* value by Chi-squared test.

**Table 2:** Time trend of completion rates in different steps of phacoemulsification performed by residents.

	Jul 2005–Sep 2007	Oct 2007–Dec 2009	Jan 2010–Mar 2012	Apr 2012–Jun 2014	Jul 2014–Sep 2016	Oct 2016–Dec 2018	Jan 2019–Mar 2021	*p* value
Total number of cases	226	201	193	237	182	178	192	
Rate in step A (cases/cases)	100%	99.5%	100%	99.6%	99.5%	100%	100%	0.705
	(226/226)	(200/201)	(193/193)	(236/237)	(181/182)	(178/178)	(192/192)	
Rate in step B (cases/cases)	80.5%	83.5%	82.9%	82.3%	89.0%	93.3%	95.8%	<0.001
	(182/226)	(167/200)	(160/193)	(195/237)	(161/181)	(166/178)	(184/192)	
Rate in step C (cases/cases)	61.1% (124/203)	75.1% (133/177)	77.6% (128/165)	70.7% (140/198)	75.9% (123/162)	82.1% (138/168)	87.0% (160/184)	<0.001
Rate in step D (cases/cases)	94.1% (176/187)	96.2% (150/156)	97.8% (132/135)	98.6% (141/143)	97.6% (121/124)	97.1% (134/138)	98.8% (161/163)	0.159
Rate in step E (cases/cases)	98.3% (178/181)	99.3%	99.2%	99.3%	99.2%	99.3%	99.4%	0.954
		(152/153)	(132/133)	(141/142)	(121/122)	(133/134)	(160/161)	

A: wound incision, B: anterior capsulorhexis, C: hydrodissection, hydrodelineation, and emulsification of nucleus, D: irrigation and aspiration of cortex, and E: IOL insertion. *p* value by Chi-squared test.

**Table 3:** Time trend of complication rates in different steps of phacoemulsification performed by residents.

	Jul 2005–Sep 2007	Oct 2007–Dec 2009	Jan 2010–Mar 2012	Apr 2012–Jun 2014	Jul 2014–Sep 2016	Oct 2016–Dec 2018	Jan 2019–Mar 2021	*p* value
Events/cases	63/226	42/201	28/193	28/237	16/182	17/178	14/192	
Rate in step A (events/cases)	0.4%	0.5%	0%	0%	0.5%	0%	0%	0.6761
	(1/226)	(1/201)	(0/193)	(0/237)	(1/182)	(0/178)	(0/192)	
Rate in step B (events/cases)	10.2%	9.5%	6.2%	6.8%	2.8%	2.2%	2.6%	0.0007
	(23/226)	(19/200)	(12/193)	(16/237)	(5/181)	(4/178)	(5/192)	
Rate in step C (events/cases)	13.3% (27/203)	9.6% (17/177)	7.9% (13/165)	5.6% (11/198)	5.6% (9/162)	6.0% (10/168)	3.8% (7/184)	0.0085
Rate in step D (events/cases)	5.3% (10/187)	3.2% (5/156)	1.5% (2/135)	0.7% (1/143)	0.8% (1/124)	2.2% (3/138)	0.6% (1/163)	0.027
Rate in step E (events/cases)	1.1% (2/186)	0%	0.8%	0%	0%	0%	0.6%	0.4103
		(0/153)	(1/133)	(0/142)	(0/122)	(0/134)	(1/161)	

A: wound incision; B: anterior capsulorhexis; C: hydrodissection, hydrodelineation, and emulsification of nucleus; D: irrigation and aspiration of cortex; and E: IOL insertion. *p* value by Chi-squared test.

## Data Availability

The underlying data are surgical records stored in the medical charts of the Chang Gung Memorial Hospital.
